# Highlights on distinctive structural and functional properties of HTLV Tax proteins

**DOI:** 10.3389/fmicb.2013.00271

**Published:** 2013-09-09

**Authors:** Maria Grazia Romanelli, Erica Diani, Elisa Bergamo, Claudio Casoli, Vincenzo Ciminale, Françoise Bex, Umberto Bertazzoni

**Affiliations:** ^1^Department of Life and Reproduction Sciences, University of VeronaVerona, Italy; ^2^GEMIB laboratory, Center for Medical Research and Molecular DiagnosticsParma, Italy; ^3^Department of Surgery, Oncology and Gastroenterology, University of PaduaPadua, Italy; ^4^Institute for Microbiological Research J-M Wiame, Laboratory of Microbiology, Université Libre de BruxellesBrussels, Belgium

**Keywords:** HTLV, Tax proteins, signal transduction, NF-κB

## Abstract

Human T cell leukemia viruses (HTLVs) are complex human retroviruses of the *Deltaretrovirus *genus. Four types have been identified thus far, with HTLV-1 and HTLV-2 much more prevalent than HTLV-3 or HTLV-4. HTLV-1 and HTLV-2 possess strictly related genomic structures, but differ significantly in pathogenicity, as HTLV-1 is the causative agent of adult T cell leukemia and of HTLV-associated myelopathy/tropical spastic paraparesis, whereas HTLV-2 is not associated with neoplasia. HTLVs code for a protein named Tax that is responsible for enhancing viral expression and drives cell transformation. Much effort has been invested to dissect the impact of Tax on signal transduction pathways and to identify functional differences between the HTLV Tax proteins that may explain the distinct oncogenic potential of HTLV-1 and HTLV-2. This review summarizes our current knowledge of Tax-1 and Tax-2 with emphasis on their structure, role in activation of the NF-κB (nuclear factor kappa-B) pathway, and interactions with host factors.

## INTRODUCTION

The Human T cell leukemia viruses (HTLVs) are complex retroviruses, belonging to the primate T-lymphotropic virus (PTLV) family. HTLVs are classified as Deltaretroviruses, together with bovine leukemia virus (BLV) and simian T-lymphotropic viruses (STLVs). HTLV-1 was originally described in 1980 (Poiesz et al., 1980) and was the first oncogenic retrovirus discovered in humans (reviewed by Gallo, 2011; Currer et al., 2012). HTLVs originated in Africa around 30,000–40,000 years ago through cross-species transmission of STLVs from monkeys to man. The virus evolved to HTLV and spread to different geographic regions with human migration (Van Dooren et al., 2001). STLVs with high homology to HTLVs are still present in Africa (Hajj et al., 2012). HTLVs are transmitted both vertically and horizontally (reviewed in Watanabe, 2011; Yasunaga and Matsuoka, 2011; Lairmore et al., 2012) but cell-to-cell transmission is essential and occurs through direct contact through the formation of a virological synapse (Nejmeddine et al., 2005; Asquith et al., 2007; Majorovits et al., 2008).

HTLV-1 has received much scientific attention due to its ability to transform primary human T-lymphocytes in cell culture and its association with a neoplasia and a neuropathology (Matsuoka and Jeang, 2007). The most important HTLV-1-associated diseases are the adult T cell leukemia (ATL), a very aggressive form of leukemia, and the HTLV-associated myelopathy/tropical spastic paraparesis (HAM/TSP), a neurological demyelinating disease (Osame et al., 1986; Bangham and Osame, 2005; Yoshida, 2010; Gessain and Mahieux, 2012; Yamagishi and Watanabe, 2012). Three additional genotypes of HTLV, named HTLV-2, HTLV-3, and HTLV-4, have been isolated and characterized, with HTLV-2 the most common after HTLV-1 (Manns and Blattner, 1991; Mahieux and Gessain, 2009). Although HTLVs share a similar genomic structure, HTLV-2 is much less pathogenic than HTLV-1 since it does not cause neoplastic disorders and is sporadically associated with cases of subacute myelopathy (Feuer and Green, 2005). Understanding the molecular basis of the different pathogenicity of HTLV-1 and HTLV-2 may thus provide important clues to the molecular mechanisms of cancer. All HTLVs possess an open reading frame (ORF) encoding the Tax transactivator, which is essential for proviral gene expression from the viral long terminal repeat (LTR) promoter and also regulates the expression and function of a number of cellular genes and proteins (Feuer and Green, 2005; Wycuff and Marriott, 2005; Calattini et al., 2006; Chevalier et al., 2006). Tax alone is capable of modulating several pathways by activating the transcription factors nuclear factor kappa-B (NF-κB) and cyclic AMP responsive binding protein (CREB; Currer et al., 2012; Tang et al., 2013a). Tax also interacts with proteins controlling cell cycle checkpoints (Akagi et al., 1996; Chlichlia and Khazaie, 2010). HTLV-1 Tax (Tax-1) is necessary and sufficient for T cell immortalization (Akagi et al., 1995) and an ATL-like syndrome has been observed in transgenic mice expressing Tax in the T cell compartment (Ohsugi, 2013). It is noteworthy to mention that in ATL patients, Tax expression is silenced in about 50% of the patients. This observation, along with the fact that Tax is capable of transforming primary T lymphocytes *in vitro*, suggests that Tax might be important for establishing the leukemic phenotype of ATL, but may become dispensable for its maintenance. In addition, recent studies suggest that other viral gene products may play relevant roles in HTLV-1-mediated transformation and may be responsible of the different HTLV-1 and HTLV-2 pathogenicity, including the antisense HTLV-1 genome transcript HBZ (Matsuoka and Jeang, 2011; Lairmore et al., 2012; Satou and Matsuoka, 2012; Zhao and Matsuoka, 2012), the HTLV-1 accessory protein p12 (Kannian and Green, 2010) and p13 (Silic-Benussi et al., 2010a, b). 

Tax-1 has been very extensively studied and its interactome includes more than 100 proteins (for a recent review see Currer et al., 2012). To further clarify this point and to better understand the reasons for the difference in pathogenicity between HTLV-1 and HTLV-2 as well as from other HTLVs, in this review we have focused on the structural and functional properties of the different Tax proteins and their relations with other viral and cellular factors. The following sections highlight recent advances in the comprehension of: (i) the transformation potential of Tax-1, Tax-2, Tax-3, and Tax-4 proteins; (ii) Tax’s role in the deregulation of signal transduction focusing on studies which describe novel interactions of Tax proteins with host factors and contribute to the understanding of the molecular mechanisms of cell response to viral infection; and (iii) Tax-mediated activation of the NF-κB pathway focusing on differences between Tax-1 and Tax-2 involvement in canonical and non-canonical pathways.

## SPECIFIC FEATURES OF DIFFERENT HTLV GENOTYPES

Following the discovery of HTLV-1 in 1980, three additional HTLVs were found: HTLV-2 in 1982 (Kalyanaraman et al., 1982; Vandamme, 2000; Gallo, 2002) and HTLV-3 and HTLV-4 in 2005 (Calattini et al., 2005; Wolfe et al., 2005). These four genotypes show specific geographical areas of distribution. HTLV-1, which includes seven subtypes (HTLV-1A to -1G), is endemic in Japan, sub-Saharan Africa, South America, the Caribbean Islands, and Melanesia. About 5–10 million people worldwide are infected with HTLV-1, most of whom are expected to remain asymptomatic throughout their lifetime (Gessain and Cassar, 2012). An estimated 2–5% of infected people develop clinical complications including ATL, HAM/TSP, infective dermatitis, uveitis, arthritis, and infection by *Strongyloides stercoralis* (Gonçalves, 2010). HTLV-2, for which the four subtypes -2A to -2D are known, is endemic within the Amerindian and Pygmy populations, and was found to be epidemic in intravenous drug users (Feuer and Green, 2005). In contrast to HTLV-1, HTLV-2 does not cause proliferative blood diseases. However, HTLV-2 has been linked to neurological disorders, arthritis, pneumonia, and with increased mortality (Araujo and Hall, 2004; Roucoux and Murphy, 2004; Biswas et al., 2010). The two new genotypes, termed HTLV-3 and HTLV-4, were discovered in asymptomatic individuals from Cameroon (Calattini et al., 2005; Wolfe et al., 2005); the pathogenic potential of these viruses is still unknown. HTLV-3 is closely related to the simian virus STLV-3, whereas an STLV corresponding to HTLV-4 has not yet been found (Sintasath et al., 2009).

**FIGURE 1:**
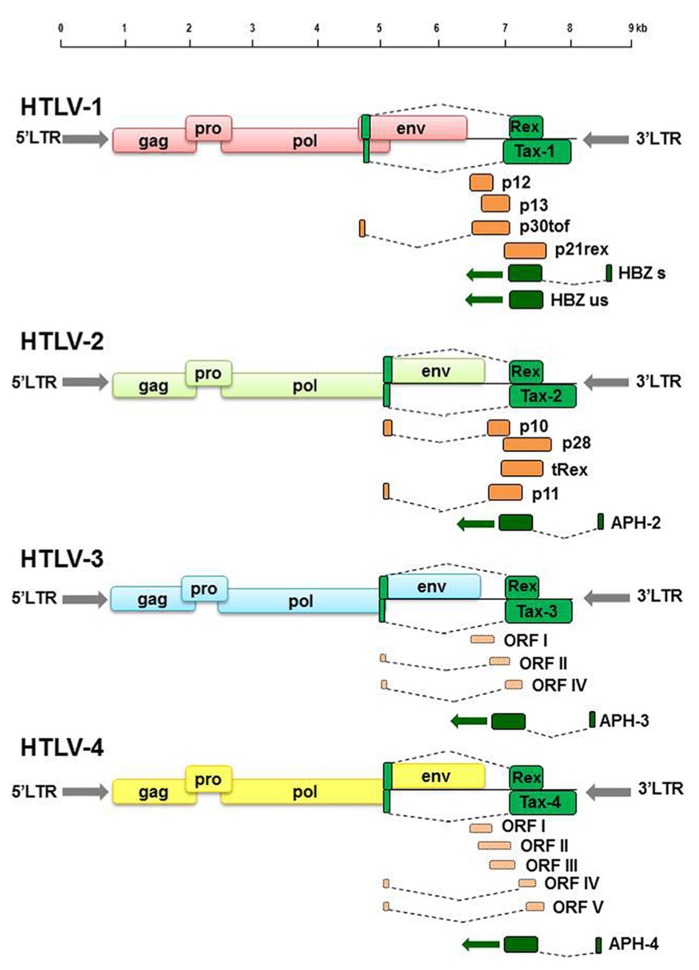
**Schematic representation of HTLV-1, HTLV-2, HTLV-3, and HTLV-4 genomic organization.** Green colored boxes indicate ORF encoding regulatory proteins. Dark orange colored boxes indicate ORF encoding auxiliary proteins. Light orange colored boxes indicate putative ORF deduced by genomic sequences analyses.

Comparative studies of the genomic sequences of all four HTLV genotypes have highlighted common as well as unique molecular features. HTLV-1 and HTLV-2 have a similar genomic structure and share approximately 70% nucleotide sequence homology (Feuer and Green, 2005). HTLV-3 and HTLV-4 have a genomic organization which is similar to that of HTLV-1 and HTLV-2 with the presence of *gag, pro, pol*, and* env* ORFs as well as of *tax *and* rex*, whereas ORFs for auxiliary proteins still need to be confirmed (Gessain et al., 2013; **Figure 1**). HTLV-3 shares about 62% identity with HTLV-1 and HTLV-4 shares 62–71% nucleotide similarity with HTLV-1, HTLV-2, and HTLV-3 (Switzer et al., 2006). HTLV-3 and HTLV-4 present LTRs that lack the distal 21 bp transcription regulatory repeat sequence (Switzer et al., 2006). Both HTLV-3 and HTLV-4 present on the antisense strand a potential ORF named APH-3 and APH-4, respectively (antisense protein of HTLV), analogous to the HBZ gene of HTLV-1 (Larocque et al., 2011) and APH-2 of HTLV-2 (Halin et al., 2009). Sequence alignment indicated that APH-3 and APH-4 are more closely related to APH-2 than to HBZ (Larocque et al., 2011). The proteins also present some differences, as APH-2, APH-3, and APH-4 do not contain a consensus bZIP (basic leucine zipper) domain present in HBZ, and differ in their subcellular localization as compared to HBZ (Halin et al., 2009; Larocque et al., 2011).

## COMPARISON OF Tax PROTEINS STRUCTURES

**FIGURE 2:**
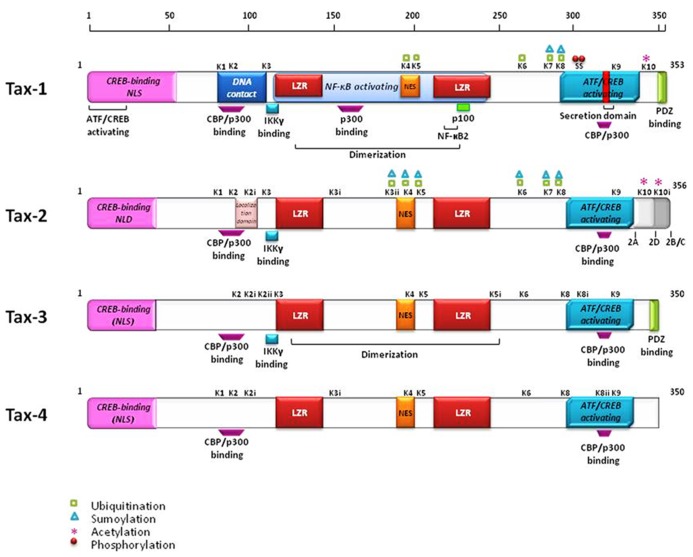
**Structural and functional domains of the Tax proteins.** The position of the lysines and their ubiquitination, sumoylation, and acetylation are indicated. Red dots identify phosphorylated serines.

**FIGURE 3:**
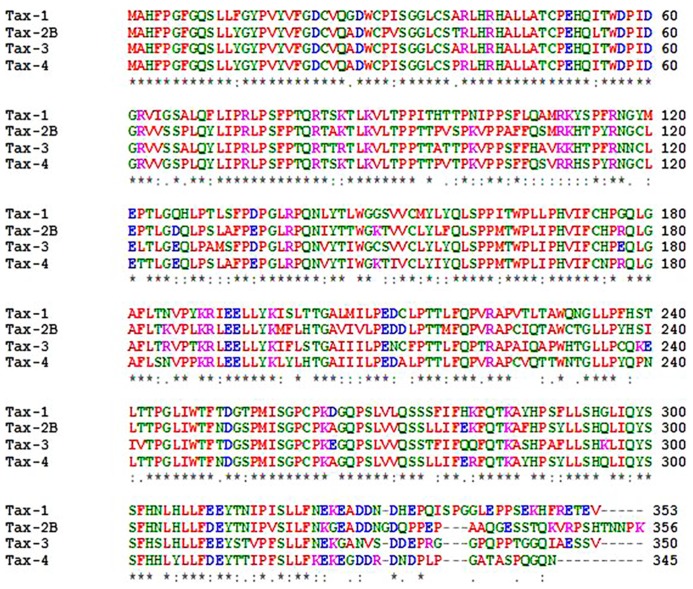
**ClustalW alignments of Tax-1, Tax-2B, Tax-3, and Tax-4 proteins**.

The *tax* gene is encoded within the pX region of HTLV, located between *env *and the 3′-LTR, and is highly conserved among all four genotypes and different subtypes of the virus (Currer et al., 2012). The Tax protein is common to all primate members of the PTLV**family. The primary amino acids (aa) sequence of Tax-1 is composed of 353 residues, and is organized into functional domains that have been extensively investigated (Bertazzoni et al., 2011; Currer et al., 2012). The structural and functional domains of Tax-1, Tax-2, Tax-3, and Tax-4 are shown in **Figure 2**. The N-terminal region of Tax-1 contains a CREB-binding region (Yin et al., 1995) that spans aa 1–60. This region is involved in the interaction with ATF/CREB transcription factors and represents a binding domain required for interaction with proteins involved in transcription, cell cycle progression, and cell signaling regulation (Suzuki et al., 1993a; Goren et al., 1995). All Tax proteins contain the CREB-binding domain within their N-terminus. A nuclear localization signal (NLS) is located within the first 60 aa in Tax-1 (Smith and Greene, 1992), Tax-3 and Tax-4 (Calattini et al., 2006; Switzer et al., 2009). A nuclear localization determinant (NLD) is present within the first 42 aa in Tax-2 (Sheehy et al., 2006; Turci et al., 2006a). An additional localization domain is attributed to Tax-2 at aa position 90–100, which confers to the protein a more abundant accumulation into the cytoplasm as compared to Tax-1 (Meertens et al., 2004a). All four Tax proteins contain a conserved region representing a nuclear export sequence (NES) that has been functionally characterized in both Tax-1 and Tax-2 and is located at aa position 189–202 in Tax-1 (Alefantis et al., 2003; Chevalier et al., 2005). Two leucine zipper-like motif regions (LZRs) are present at aa 116–145 and 213–248 in Tax-1 and conserved in Tax-2, Tax-3, and Tax-4 as well. These regions are required for protein dimerization and binding of cellular factors (Jin and Jeang, 1997; Basbous et al., 2003). Tax-1 and Tax-3 are characterized by the presence of a PDZ-binding motif (PBM) at the C-terminal region (Chevalier et al., 2006) whereas this is missing in Tax-2 and Tax-4. This domain is required for interactions between Tax-1 and cellular factors such as the tumor suppressors hDlg, MAGI-1, and Scribble and the synapse-associated protein DlgI (Rousset et al., 1998; Suzuki et al., 1999; Okajima et al., 2008; Yoshida et al., 2008; Makokha et al., 2013). The absence of the PDZ domain renders Tax-2 unable to interact with these factors. The four subtypes of HTLV-2 (-2A to -2D) code for similar but not identical Tax proteins. Tax-2A is composed of 331 aa and is shorter than Tax-2B, Tax-2C, and Tax-2D (356, 356, and 344 aa, respectively; Feuer and Green, 2005), Tax-2B is the variant that has been studied in greatest detail (Bertazzoni et al., 2011). Tax-1 and Tax-2B share 85% aa similarity, whereas Tax-3 displays 26 and 30% divergence with respect to Tax-1 and Tax-2, respectively. The comparison of Tax-4 with Tax-1, Tax-2, and Tax-3 shows 83, 91, and 85% aa similarity, respectively (Switzer et al., 2009), as outlined in **Figure 3**. The main characteristic that distinguishes Tax-1 from Tax-2 is the presence only in Tax-1 of a motif spanning aa 225–232 that activates the non-canonical NF-κB pathway through interaction with the p100 factor (Shoji et al., 2009). A second relevant difference between Tax-1 and Tax-2 is the presence in Tax-1, but not in Tax-2, of the PBM at the C-terminus (Higuchi and Fujii, 2009; Bertazzoni et al., 2011; Rende et al., 2012). Based on sequence homology, all the Tax proteins possess two functional regions involved in CBP (CREB-binding protein)/p300 binding: a KID-like domain between residues 81 and 95 and a second domain named C-terminal transcriptional activating CR2 domain, between aa 312 and 319. These domains present some differences between Tax-1, Tax-2, Tax-3, and Tax-4. One of the major differences is related to the lysine residue at position 85, which is necessary for Tax-1 to bind CBP/p300 (Hiramatsu and Yoshikura, 1986); this residue is substituted by an arginine in Tax-2, Tax-3, and Tax-4 (Calattini et al., 2006; Chevalier et al., 2006).

Post-translational modifications of Tax-1 and Tax-2 such as phosphorylation, acetylation, ubiquitination, and sumoylation have been extensively described in recent reviews (Bertazzoni et al., 2011; Lodewick et al., 2011; Kfoury et al., 2012). Recent comparative analyses of Tax-1 and Tax-2 post-translational modifications centered on the contribution of ubiquitination and sumoylation to the intracellular localization of Tax and its ability to activate NF-κB (Lamsoul et al., 2005; Kfoury et al., 2006; Nasr et al., 2006; Turci et al., 2009, 2012; Bonnet et al., 2012; Xiao, 2012; Zane and Jeang, 2012; Journo et al., 2013). These studies highlighted the role of specific lysines as targets for sumoylation and ubiquitination in Tax-1 and Tax-2 (lysines K1 to K10 in Tax-1, see **Figure 2**). Lysines K6 and K8 are critical for NF-κB activation and are highly conserved in all Tax proteins. Additional lysines, indicated by additional roman numbers, are present in Tax-2 (K2i, K3i, K3ii, and K10i, **Figure 2**) whereas Tax-3 and Tax-4 contain 11 and 10 lysines, respectively, but not all are conserved at the same position. Alignment of the predicted Tax-4 sequence shows the absence of K10, which is a target of acetylation in Tax-1 and possibly in Tax-2 (Lodewick et al., 2009; Journo et al., 2013).

Tax is generally defined as a multifunctional protein, since it is able to activate both viral and host gene transcription and to act as mediator of several cellular pathways. Of particular importance was the demonstration that Tax-1 is able to immortalize and transform human primary CD4^+^ T cells (Grassmann et al., 1992; Franchini, 1995; Yoshida, 2001) and the finding that Tax-1-transgenic mice develop ATL (Hasegawa et al., 2006; Ohsugi, 2013). It is well-established that Tax-1 plays a relevant role in the oncogenesis induced by HTLV infection. Tax-1 acts as a pleiotropic protein conferring proliferative and survival properties to HTLV-1 – infected cells by modulating regulatory factors that induce cell proliferation, cell cycle progression, inhibition of apoptosis, and interference with DNA repair. The main factors include CREB, CBP/p300, NF-κB, cyclin-dependent kinases (CDKs), and Akt (reviewed by Kannian and Green, 2010; Currer et al., 2012).

## TRANSFORMATION POTENTIAL OF Tax PROTEINS

Tax-1 is able to transform T-lymphocytes and fibroblasts and to induce tumors in transgenic mice (Azran et al., 2004; Matsuoka and Jeang, 2007; Kannian and Green, 2010; Ohsugi, 2013). The ability to transform primary human T cells was also demonstrated for Tax-2 (Ross et al., 1996; Feuer and Green, 2005). Both proteins are able to inhibit the function of the tumor suppressor p53. A comparative study between Tax-1 and Tax-2 subtypes demonstrated that Tax-2A inhibits p53 function less efficiently than Tax-1 or Tax-2B (Mahieux et al., 2000). Although several *in vitro* studies have investigated the ability of Tax to inactivate p53 (Tabakin-Fix et al., 2006) by acting on the CREB or NF-κB pathway, the mechanism of this inhibition has not yet been completely clarified. A recent study by Zane et al. (2012) identified a cooperative role for the cellular factor Wip1 (wild-type p53-induced phosphatase 1) in Tax-1-mediated inactivation of p53; a subsequent study demonstrated interactions between Tax-1 and Wip1 (Dayaram et al., 2013). In a study that employed transgenic mice expressing Tax-1 which develop mature T cell leukemia and lymphoma, Ohsugi et al. (2013) demonstrated that Tax-1 alters p53 function and that this effect precedes NF-κB activation.

Tax-2 transforms rat fibroblasts less efficiently than Tax-1 (Endo et al., 2002). On the other hand, both viruses immortalizes primary human T cells at a comparable efficiency (Feuer and Green, 2005). Higuchi et al. (2007) demonstrated that the non-canonical NF-κB factor p100 and the PBM present in Tax-1, but not in Tax-2, are essential for the transformation of a T cell. Tsubata et al. (2005) had already shown that the PBM domain is critical for the ability of Tax-1 to induce interleukin-2 (IL-2)-independent growth of the IL-2-dependent T cell line CTLL-2, and that Tax-2 lacks this ability. Xie et al. (2006) showed that the deletion of the PBM in a recombinant HTLV-1 molecular clone (HTLV-1/ΔPBM) alters the requirement for the establishment and maintenance of persistent infection in rabbits. A motif responsible for the distinct transforming activity of Tax-1 and Tax-2 was identified by using a series of Tax-1/Tax-2 chimeric proteins. A region corresponding to aa 225–232 of Tax-1 was shown to play a crucial role in Tax-1’s transforming activity, involving stimulation of the non-canonical NF-κB/p100 pathway (Shoji et al., 2009). Imai et al. (2013) recently demonstrated that Tax-2B can immortalize human CD4^+^ T cells. By infecting peripheral blood mononuclear cells (PBMCs) with lentiviruses encoding Tax-1 or Tax-2B they observed a higher immortalization activity of Tax-2B as compared to Tax-1.

Studies of Tax-2-immortalized T cells demonstrated that Tax-2 causes a dysregulation of autophagy; this may represent a novel survival mechanism in Tax-2-immortalized T cells (Ren et al., 2012). A similar action was attributed to Tax-1, thus suggesting that autophagy may play an important role in the HTLV life cycle (Tang et al., 2013b). Tax-3 was shown to be able to activate the NF-κB pathway and bind CBP in the T cell line CEM, thus suggesting that Tax-3 has *in vitro* transforming activity (Chevalier et al., 2006). The transforming properties of Tax-4 remain to be investigated.

## Tax AND SIGNAL TRANSDUCTION DEREGULATION

The role of Tax in HTLV-1-induced oncogenesis has been investigated in large part by analyzing the capacity of Tax-1 to interact with selected cellular factors that play a crucial role in signaling pathways. A list of Tax-interacting proteins is presented in **Table 1**. Tax-1 expression deregulates several signaling pathways involved in the cell cycle, cell proliferation, and cell survival, primarily through the deregulation of two major cellular transcription factor pathways: CREB/ATF and NF-κB (Sun and Yamaoka, 2005; Nyborg et al., 2010). Tax-1 constitutively activates NF-κB by causing a deregulated expression of a variety of cellular genes. Tax-dependent NF-κB activation has been extensively studied and the current state of knowledge will be described in the next section. Tax-1 activation through the cellular transcription factor CREB has been well-characterized at the level of the HTLV-1 promoter located in the LTR region. Within the HTLV-1 promoter three conserved 21 bp repeat enhancer elements called viral CRE elements (vCRE) are present that can be recognized within a complex containing Tax-1 and a phosphorylated form of CREB. The Tax/CREB/vCREB complexes can be associated to other host factors. The best characterized are the cellular coactivators CBP and p300 (Kashanchi and Brady, 2005), which stimulate Tax-mediated transactivation by chromatin remodeling (Nyborg et al., 2010). In addition, Tax-1 interacts with the SWI/SNF chromatin remodeling complexes (Easley et al., 2010) and may be involved in the nucleosome eviction activity mediated by the nucleosome assembly protein 1 (NAP1; Sharma and Nyborg, 2008). Additional host factors that directly interact with Tax-1 and act in the Tax-mediated transactivation are the transducer of regulated CREB (TORC) proteins. TORC-1 and TORC-2 are required for Tax activation whereas TORC-3 enhances Tax-dependent transcription (Koga et al., 2004; Siu et al., 2006). Several cellular factors that interact with Tax and participate to HTLV-1 promoter activation have been identified. The transcriptional activator CIITA affects the functional interaction of the transcription factors CREB, ATF1, and PCAF with Tax-1 (Tosi et al., 2011) and Tax-2 activation of HTLV-2 LTR is strongly inhibited by CIITA (Orlandi et al., 2011). Recently, Tang et al. (2013a) demonstrated that the LKB1 tumor suppressor and the salt inducible kinases (SIKs) act as negative regulatory factors in the activation of HTLV-1 LTR by Tax. They showed that LKB1 and SIK interact with Tax and that this association enables LTR activation by TORCs, CREB, and Tax-1 (Tang et al., 2013a). Additional cellular mediators of Tax-induced activation of HTLV-1 LTR belong to the group I p21-activated kinases (Paks) which physically interact with Tax and CREB-regulating transcriptional coactivators to facilitate HTLV-1 transcription (Chan et al., 2013).

**Table 1:** Tax-1 interacting proteins and deregulated pathways.

Pathways	Factors	Reference
G proteins	Gβ subunit	Twizere etal. (2007)
	Rho GTPases	Wu etal. (2004)
MAPKs	MEKK1	Yin etal. (1998)
	TAK1	Wu and Sun (2007)
JNK	GPS2	Jin etal. (1997)
AP1	p85α	Peloponese and Jeang (2006)
TGFβ	Smad2	Mori etal. (2001)
	Smad3	
	Smad4	
NF-κB	IKKα	Chu et al. (1998)
	IKKβ	
	IKKγ/NEMO	Harhaj and Sun (1999)
	IkBα	Suzuki etal. (1995)
	IkBγ	Hirai etal. (1994)
	RelA	Suzuki etal. (1994)
	p100	Béraud etal. (1994)
	p50	Suzuki etal. (1993b)
	TAK	Wu and Sun (2007)
	TRAF6	Journo etal. (2013)
	NRP/optineurin	Journo etal. (2009)
	USP20	Yasunaga etal. (2011)
	TAX1BP1	Journo etal. (2009)
CREB	CBP/p300	Kwok etal. (1996)
	CREM	Suzuki etal. (1993a)
	ATF-4	Reddy etal. (1997)
	XBP-1	Ku etal. (2008)
	TORC	Koga etal. (2004)
	LKB1	Tang etal. (2013a)
	SIK1	Tang etal. (2013b)
	Paks	Chan etal. (2013)
	CIITA	Tosi etal. (2011)
SW1/SNF	PBAF	Easley etal. (2010)
HDAC	CBP/p300	Ego et al. (2002)
Histone modification	CARM1/SMYD3	Yamamoto etal. (2011)
SRF	SRF	Fujii etal. (1992)
	Elk-1	Shuh and Derse (2000)
	SAP-1	
Microtubule formation	TAB2	Yu etal. (2008)
	hsMAD1/TXBP181	Jin etal. (1998)
	Tax1BP2	Ching etal. (2006)
G1/S transition	Cdk4	Haller etal. (2002)
	Cdk6	
DNA repair	DNA-PK	Durkin etal. (2008)
	ATM	Dayaram etal. (2013)
	CHK2	
	Wip1	
PI3K/Akt1 signaling	Beclin1	Cheng etal. (2012)
	PI3KC3	
Tumor suppression	MAGI-1	Makokha etal. (2013)
	Scribble	Okajima etal. (2008)
	hDlg	Suzuki etal. (1999)

The role of Tax-1 in the expression of cellular genes containing CRE elements was demonstrated for several genes involved in cell cycle and proliferation. Recently Kim et al. (2010) demonstrated that Tax-1 deregulates cyclin D1 gene expression thus determining its overexpression. The mechanism requires an enhanced binding between p300 and phosphorylated CREB and TORC-2. The interaction of Tax-1 with CREB/ATF factors also represses the expression of several genes, including cyclin A (Kibler and Jeang, 2001), p53 (Mulloy et al., 1998), c-myc (Semmes et al., 1996a), and the *ZNF268 *gene, which**plays a role in the differentiation of blood cells during development and in the pathogenesis of leukemia (Wang et al., 2008).

The main functional and structural differences between Tax-1, Tax-2, and Tax-3 are presented in **Table 2**. It is evident that the Tax proteins differ not only in their transformation abilities, structural properties, and protein interactions, as described in the previous sections, but also in additional aspects of cellular interactions. Tax-2 is distributed both in the nucleus and in the cytoplasm, showing a more diffuse distribution in the cytoplasm compared to Tax-1 (Turci et al., 2009). We have recently demonstrated that Tax-1 and Tax-2 colocalize with TAB2-containing cytoplasmic structures that include RelA and calreticulin (Avesani et al., 2010). Compared to Tax-1, Tax-2 differs in post-transcriptional modification (Lodewick et al., 2011) and we have shown that, in transfected cells, lysine usage for sumoylation differs between Tax-1 and Tax-2 (Turci et al., 2012). When compared to Tax-1, Tax-2 is less efficient in the induction of micronuclei formation (Semmes et al., 1996b), is unable to suppress multilineage hematopoiesis from CD34^+^ cells *in vitro* (Tripp et al., 2003) and to direct the lipid raft translocation of IκB kinase alpha (IKKα) and IKKβ in transfected cells and in Tax-2-immortalized primary T cells (Huang et al., 2009).

**Table 2:** Summary of main functional and structural differences between Tax-1, Tax-2, and Tax-3.

	Tax-1	Tax-2^a^	Tax-3	Reference
Transactivating activity	Higher^a^	Lower^b^	n.d.^c^	Semmes etal. (1996a)
Transformation capacity	Higher	Lower	n.d.	Endo etal. (2002)
Micronuclei formation	+	-	n.d.	Semmes etal. (1996b)
Cell cycle arrest	+	-	n.d.	Tripp etal. (2005)
Hematopoiesis suppression	+	-	n.d.	Tripp etal. (2003)
Reduction of histone gene expression	+	-	n.d.	Harrod et al. (2000);Ego et al. (2002)
Inhibition of p53 functions	Higher	Lower	+	Mahieux et al. (2000);Meertens et al. (2004b),Jeong et al. (2005);Calattini et al. (2006)
Total viral mRNA expression	Higher	Lower	n.d.	Li and Green (2007)
Proinflammatory cytokine expression	Higher	Lower	n.d.	Banerjee etal. (2007)
Presence of PDZ motif	+	-	n.d.	Feuer and Green (2005)
Interaction with PDZ-binding proteins	+	-	+	Higuchi and Fujii (2009)
Interaction with p100	+	-	n.d.	Shoji etal. (2009)
Preferential cellular localization	Nucleus	Cytoplasm	n.d.	Turci etal. (2009)
NF-κB transactivation	+	+	+	Chevalier etal. (2012)
NF-κB transactivation (lipid raft translocation of IKK)	+	-	n.d.	Huang etal. (2009)
*In vitro* CK2 phosphorylation	+	-	n.d.	Bidoia etal. (2010)
Oligo-sumoylation	+	-	n.d.	Turci etal. (2009)
Nuclear bodies	Larger	Smaller	n.d.	Turci etal. (2009)
Ubiquitination and sumoylation	+	+	n.d.	Turci etal. (2012);Zane etal. (2012)
Nuclear localization	+	+	+	Calattini etal. (2006)
T cell immortalization	+	+	+	Chevalier et al. (2006); Imai et al. (2013)

a The properties of Tax-2 include those described for Tax-2A and/or Tax-2B reported in the literature.

b Higher and lower refers to a comparison between Tax-1 and Tax-2.

c n.d.: not determined.

Recent reports have investigated the different role of Tax-1 and Tax-2 in innate immunity. We have previously demonstrated that HIV-1/HTLV-2 coinfection in drug users is associated to a delayed progression of AIDS (Turci et al., 2006b). CC-chemokines are produced spontaneously by T lymphocytes of HIV-1/HTLV-2 coinfected subjects (Lewis et al., 2000). Recently, it has been demonstrated that Tax-1 and Tax-2 induce the expression of the CC-chemokines MIP1-α/CCL 3 MIP-1β/CCL4, and RANTES/CCL5 and that downregulate CCR5 in monocytes and PBMCs (Barrios et al., 2011; Balistrieri et al., 2013). Furthermore, a significant decrease of HIV-1 replication has been reported in cultures of PMBCs infected by HIV-1 and treated with Tax-1 or Tax-2 (Barrios et al., 2013). In this *in vitro* cell system the effect of Tax-2 on HIV-1 replication is higher than that of Tax-1 and the authors suggest that Tax-2 may act as an immunomodulatory protein during HTLV-2 infection.

An emerging role in HTLV-1 pathogenesis is attributed to the antisense protein HBZ which contains a bZIP motif, required to form heterodimers with cellular transcription factors. HBZ inhibits viral and cellular expression by interacting with CREB and additional transcription factors and, in contrast to Tax-1, is consistently expressed in ATL cells (Matsuoka and Green, 2009). Compared to HTLV-1, HTLV-2 expresses an antisense protein named APH-2, which is structurally different from HBZ, lacking the classical bZIP domain. APH-2 is able to interact with CREB and to repress the activation of HTLV-2 gene expression mediated by Tax-2 (Halin et al., 2009). A recent study has shown that APH-2 may interact with Tax-2 and when co-expressed with Tax-2, impairs the ability of Tax-2 to activate AP-1 transcription (Marban et al., 2012). AP-1 pathway involves several factors, including Jun, Fos, Maf, and ATF that act on cell proliferation, apoptosis, and oncogenic transformation (Shaulian and Karin, 2001). The distinct structural and functional diversities of HBZ and APH-2 and their interactions with Tax proteins may be relevant for the different pathogenicity of HTLV-1 and HTLV-2 and the mechanisms need to be further investigated.

## Tax AND THE NF-κB PATHWAY

**FIGURE 4:**
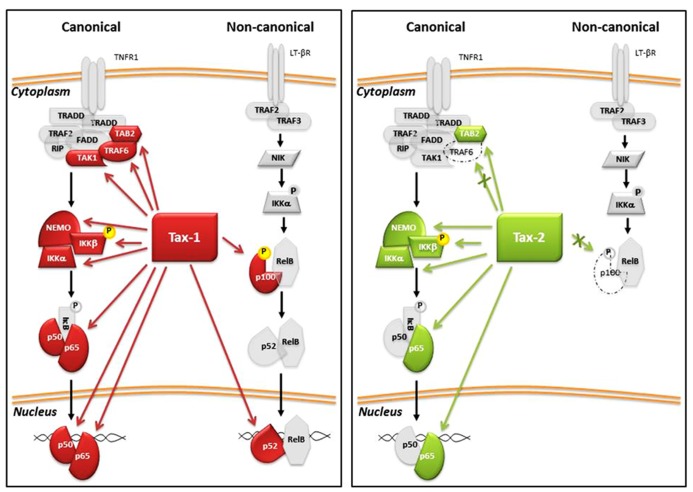
**Tax-1 and Tax-2 interactions with factors of NF-κB pathway.** Red arrows indicate Tax-1 interaction and the green arrows indicate Tax-2 interactions. Dashed proteins indicate the absence of interaction with Tax-2.

Enhanced NF-κB activation is one of the principal consequences of the expression of Tax in the infected cells. The NF-κB family of inducible transcription factors regulate diverse biological processes, including the growth and survival of both T cells and non-lymphoid cells. Activation of NF-κB transcription factors occurs through two tightly controlled signaling processes known as the canonical and non-canonical NF-κB pathways. The principal activators and regulators of these two pathways are illustrated in **Figure 4**. The canonical pathway is activated by different receptor signals including inflammatory cytokines, genotoxic stress, antigens, and toll like receptors (TLRs), whereas the activation of the non-canonical pathway involves signaling molecules that are recognized only by a specific subset of tumor necrosis factor receptors (TNFRs), such as lymphotoxin-β, BAFF, RANKL, and TWEAK (Sun, 2011). The NF-κB transcription factors family includes five members: RelA/p65, c-Rel, RelB, p50, and p52. The p50 and p52 proteins are expressed as precursor proteins named p105 and p100, respectively. The processing of these precursors to mature forms requires proteasome activity. The five members form dimers with one another and can bind to a variety of target DNA sequences called κB sites to modulate gene expression. p50 and p52 can activate transcription by forming heterodimers with RelA/p65, c-Rel, or RelB. In the cytoplasm, the NF-κB complexes are inactive since they are bound to inhibitory IκB proteins (IκBα, IκBβ, IκB∊, etc.). Activation of the pathway requires IκB protein degradation and translocation of NF-κB dimers to the nucleus. The common step of activation is mediated by the IKK complex, which phosphorylates IκB and targets it to proteosomal degradation. The IKK complex consists of two active kinases, IKKα and IKKβ, and the regulatory scaffolding protein NEMO (IKKγ). Tax directly interacts with these factors, leading to a persistent activation of NF-κB-mediated transcription. Tax-1 stimulates the activation of both the canonical and non-canonical NF-κB pathway thought the interaction with the IKK factors; the Tax/IKKγ interaction is required for recruiting Tax to the IKK catalytic subunits and for Tax-mediated IKK activation (Sun and Yamaoka, 2005).

Other cellular proteins are important for Tax-mediated NF-κB activation. Tax-1 interacts with NRP/optineurin and TAX1BP1 (Journo et al., 2009; Shembade et al., 2011), and with the ubiquitin-specific peptidase USP20 (Yasunaga et al., 2011). A recent study demonstrated that Tax-1 promotes Bcl-3 expression and nuclear translocation of RelA/p65 (Gao et al., 2013). Kim et al. (2008) provided evidence that Tax-1 induces Bcl-3 expression primarily through activation of the NF-κB pathway. Another recent study has demonstrated that Tax-1 transactivates CD69, a marker of early activation of lymphocytes, through both NF-κB and CREB signaling pathways (Ishikawa et al., 2013).

Tax-1-mediated activation of the non-canonical NF-κB pathway is important in virus-induced tumorigenesis. A region of Tax-1 spanning aa 225–232 is essential for activation of the non-canonical pathway. In contrast to Tax-1, Tax-2 is not able to activate the non-canonical pathway and does not interact with or induce processing of p100 into p52 (Higuchi et al., 2007; Shoji et al., 2009). Furthermore, Tax-2 has been demonstrated to not be able to interact with TRAF6, a protein with E3 ligase activity that, in the presence of Tax-1, positively regulates the activation of NF-κB pathway (Journo et al., 2013).

Ubiquitination and sumoylation of Tax-1 and Tax-2 are involved in NF-κB activation (Lamsoul et al., 2005; Kfoury et al., 2006; Nasr et al., 2006; Turci et al., 2009, 2012; Journo et al., 2013). Both Tax proteins co-immunoprecipitate and colocalize with IKKγ/NEMO, TAB2, and RelA/p65 in transfected cells (Meertens et al., 2004a; Sun and Yamaoka, 2005; Avesani et al., 2010). Expression of Tax proteins induces IKKα and RelA/p65 nuclear translocation (Higuchi et al., 2007; Ho et al., 2012). An overactivation of NF-κB by Tax induces cellular senescence (Zhi et al., 2011). By knockdown experiments, Ho et al. (2012) demonstrated that chronic activation of NF-κB by Tax-1 results in rapid senescence (Tax-induced rapid senescence, Tax-IRS) that is dependent on IKKα and p65/RelA activation. The Tax-IRS phenomenon constitutes a host checkpoint response to the overactivation of NF-κB that prevents cellular transformation (Zhi et al., 2011; Ho et al., 2012) and represents an interesting mechanism of host cell protection from the deregulating activities of viral proteins.

## CONCLUSION

HTLV-1 and HTLV-2 can efficiently transform T-lymphocytes, but only HTLV-1 causes ATL. Although additional viral products play important roles in the HTLV pathogenesis, Tax represents a key factor in the early stage of T cell oncogenesis. In this review we have dissected the structural and functional features of the HTLV Tax proteins, focusing mainly on Tax-1 and Tax-2. These two proteins share many common properties including the capacity of transforming and immortalizing T cells, of transactivating NF-κB pathway and being modified by both ubiquitination and sumoylation. They significantly differ for the presence of a PDZ motif, which is missing in Tax-2, and for the activation of non-canonical NF-κB which is attributed only to Tax-1. The knowledge derived by studying Tax’s interactions with cellular factors and their effects on the induction of altered responses in cell pathway regulation confirms the complexity of HTLV oncogenesis. An interesting issue that needs to be explored in the future is the frequent downregulation of the expression of viral genes coded by the plus-strand (including Tax) in circulating leukemic cells from ATL patients. This phenomenon is likely due to epigenetic silencing of the plus-strand promoter, e.g., by methylation and/or expression of repressors of the Polycomb family (Satou and Matsuoka, 2012, 2013; Yamagishi and Watanabe, 2012). A pivotal mechanism that involves a circuit controlled signaling by miRNA has been recently demonstrated by Yamagishi et al. (2012) showing that miR-31 loss, in ATL primary cells, mediated by Polycomb-dependent epigenetic gene silencing, is associated to the overexpression of the NF-κB inducing kinase NIK and leads to constitutive activation of NF-κB oncogenic signaling.

It is likely that further studies aimed at dissecting the functional differences between the Tax proteins will reveal novel functions of host factors that are involved in the signal pathways altered in ATL and may become potential targets for effective therapies against leukemia.

The studies of the differences between Tax-1, Tax-2, Tax-3, and Tax-4 interacting cell factors and transactivating activities will provide useful information to the understating of Tax-1 structural transformation that may open a new approach on HTLV studies based on Tax-1 peculiarities and interactions with addition viral products, that are not present in HTLV-2, HTLV-3, and HTLV-4 Tax proteins.

## Conflict of Interest Statement

The authors declare that the research was conducted in the absence of any commercial or financial relationships that could be construed as a potential conflict of interest.
